# Implementation of Obstacle-Avoidance Control for an Autonomous Omni-Directional Mobile Robot Based on Extension Theory

**DOI:** 10.3390/s121013947

**Published:** 2012-10-16

**Authors:** Neng-Sheng Pai, Hung-Hui Hsieh, Yi-Chung Lai

**Affiliations:** Department of Electrical Engineering, National Chin-Yi University of Technology, Taichung 41170, Taiwan; E-Mails: s4a012125@ncut.edu.tw (H.-H.H.); s4a012102@ncut.edu.tw (Y.-C.L.)

**Keywords:** extension theory, fuzzy theory, obstacle-avoidance, omni-directional mobile robot, ultrasonic sensors

## Abstract

The paper demonstrates a following robot with omni-directional wheels, which is able to take action to avoid obstacles. The robot design is based on both fuzzy and extension theory. Fuzzy theory was applied to tune the PMW signal of the motor revolution, and correct path deviation issues encountered when the robot is moving. Extension theory was used to build a robot obstacle-avoidance model. Various mobile models were developed to handle different types of obstacles. The ultrasonic distance sensors mounted on the robot were used to estimate the distance to obstacles. If an obstacle is encountered, the correlation function is evaluated and the robot avoids the obstacle autonomously using the most appropriate mode. The effectiveness of the proposed approach was verified through several tracking experiments, which demonstrates the feasibility of a fuzzy path tracker as well as the extensible collision avoidance system.

## 1. Introduction

The development of omni-directional wheel systems has made it possible to build robots that can move laterally without needing to rotate. Several researchers have employed omni-directional wheels in their robot designs. A dynamic model and a nonlinear mobile issue are explored in [1–3], where an omni-directional vehicle is equipped with up to three motor sets. Using a Field Programmable Gate Array (FPGA) as the control core, a multi-robot [4] was developed by integrating a robot arm into an omni-directional mobile robot, enabling better interaction between the robot and users.

Many factors can cause path deviation in a robot, such as variation in motor mechanical tolerances, power output, the weight borne by wheels, and even the ground surface; thus, path deviation is unavoidable. This study employs a compensation approach based on a motor encoder using fuzzy logic to resolve the problem of straight path deviation [5,6]. Motor revolutions are evaluated based on the feedback pulses that are dispatched from the motor encoder at specific intervals interval; in order to tune the value of the Pulse Width Modulation (PWM), and thus, the specified motor revolutions are set.

Several approaches have been employed to avoid obstacles. These include lasers and infrared [7,8], vision systems [9,10], and wall following using ultrasonic sensors [11–13]. Extension theory was proposed in 1983 to solve contradictions and incompatibility problems. It consists of two parts—matter-element model and extended set theory—and can be applied to classification or fault diagnosis [14]. Fuzzy theory, proposed by L. A. Zadeh in 1965, uses fuzzy rules and fuzzy inference to replace complicated equations. It's widely used in robot control [15]. In this paper, the obstacle-avoidance system is modeled as multi-dimensional obstacle-avoidance matter-elements, where the names of the extension matter-elements are the same as the number of obstacle-avoidance modes. The proposed approach utilizes ultrasound to complete the task and to implement the matter-element extension model. In this way, obstacles can be modeled and an optimal tracking approach is implemented.

This paper is organized as follows: Section 2 describes the hardware configuration of the robots. Section 3 introduces the omni-directional wheeled mobile robot designed using fuzzy logic theory. Section 4 presents an extension theory based on an obstacle avoidance system. Section 5 gives a discussion of experimental results and Section 6 presents our conclusions.

## 2. Hardware Design

**Figure 1.**
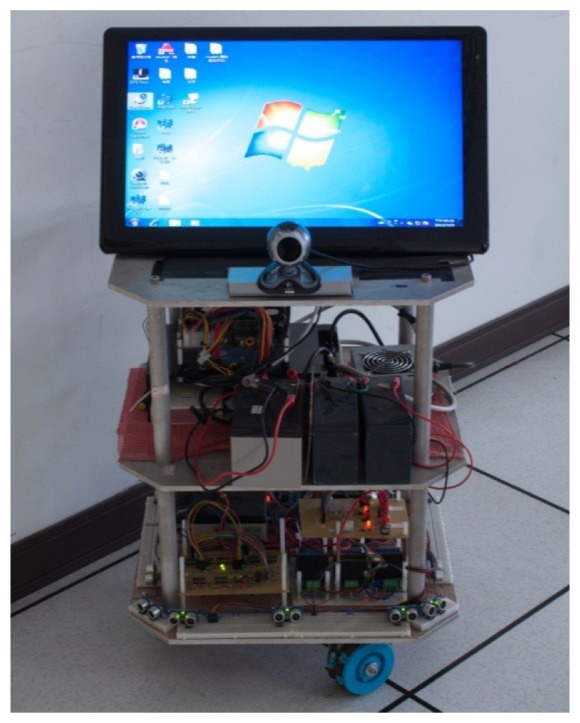
Omni-directional mobile robot.

**Figure 2.**
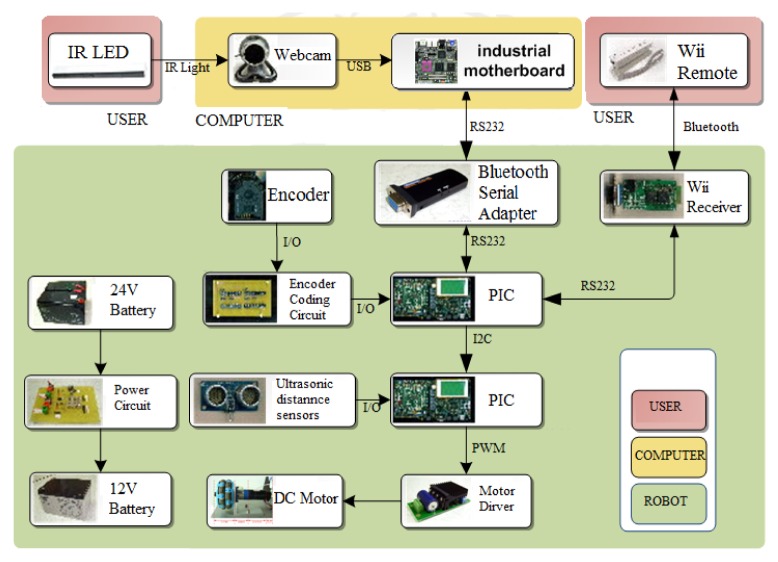
The robot system hardware link.

The omni-directional mobile autonomous following robot control system is composed of an industrial motherboard (SBC86850) along with a Peripheral Interface Controller (PIC) micro controller (DSPIC30F6010A) that serves as the control core and commands the peripheral hardware. The peripheral hardware itself consists of a motor (3863A024C), a motor driver (MD03), a motor encoder (HEDS-5500 A12), an ultrasonic distance sensor (the PING™ Ultrasonic Range Finder), and a Bluetooth controller. Figure 1 shows a diagram of the omni-directional mobile robot. Figure 2 shows the three parts of the robot tracking system, namely the user interface, a workstation, and the robots.

**Figure 3.**
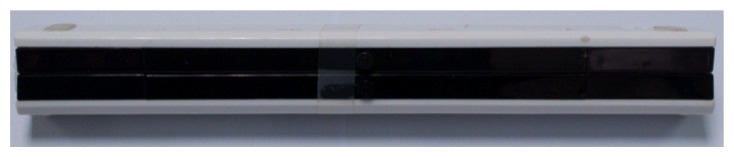
The IR source carried by the user.

There are two user modes available: manual and autonomous tracking. In manual mode, we use a Wii controller to control the robot's direction of movement and speed. In the autonomous tracking mode, the operator is provided with an infrared emitter module, as shown in Figure 3, which emits infrared signals to enable the robot to track and follow the operator autonomously.

The camera is connected to the workstation and has an infrared filter so that an infrared image is displayed on the screen. The camera is a Logitech Quick Cam Pro 5000, which captures an infrared light source handled by the operator. The industrial motherboard comprises the follower-tracking component of the system, and analyzes the infrared data, which is converted to physical coordinates giving the relative position of the user and the robot. This data is then used to control the direction and speed of the robot.

The robot employs two PIC micro controller boards, with one serving as a signal capture board and the other as a motor control board. This decentralized setup can improve the processing efficiency of the PIC micro controller. The signal capture board receives the command issued by the workstation and the Wii controller signal. The motor control board handles the motor control function, and receives information from the ultrasonic distance sensor.

## 3. An Omni-Motion Control System Based Upon Fuzzy Theory

**Figure 4.**
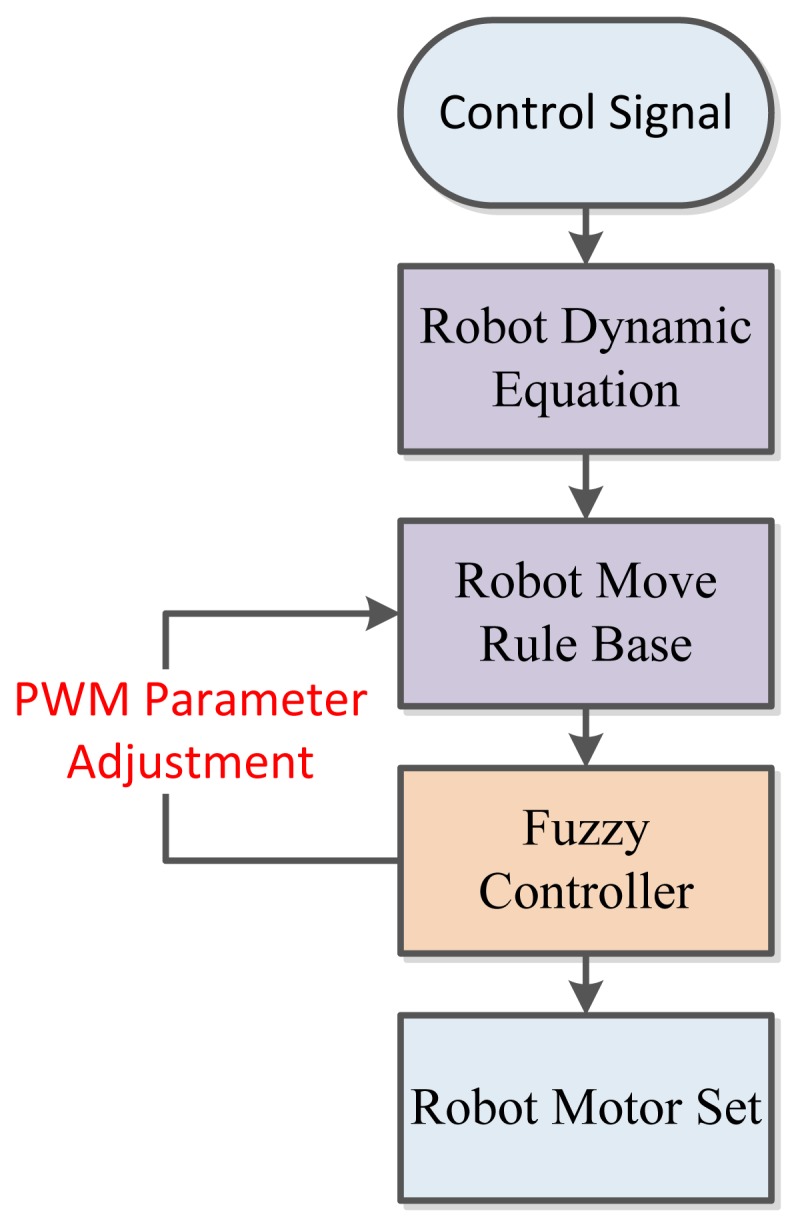
Proposed system flowchart.

The robot implemented in this research is capable of directed translation movement along the x and y axis, and rotational movement along the z axis. All signals dispatched from the motor encoders are translated into the PWM format and are used to calculate the motor revolution. These are then compensated by applying fuzzy logic theory to correct the path deviation. The flowchart of the proposed omni-motion control system is shown in Figure 4.

### 3.1. Building a Kinematic Equation

**Figure 5.**
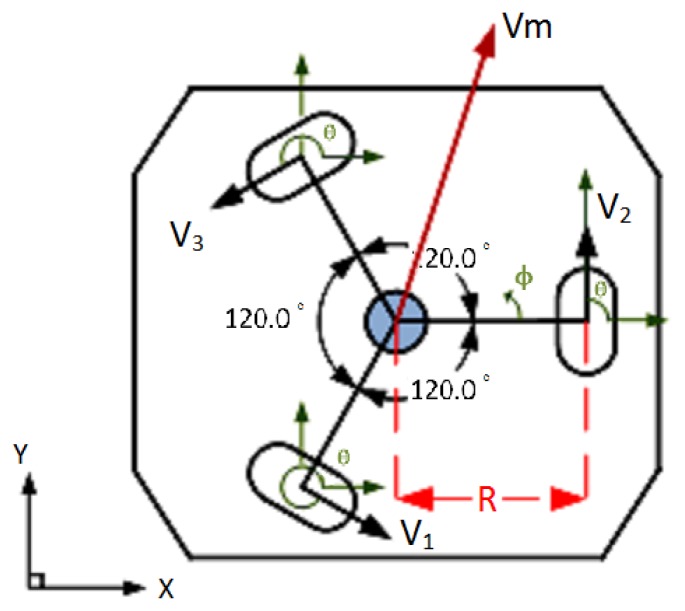
Configuration of the omni-directional mobile robot.

**Figure 6.**
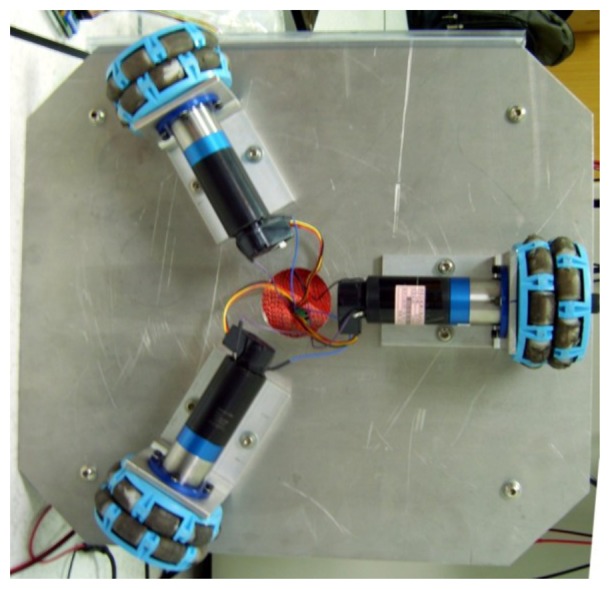
The experimental setup of the omni-directional mobile robot.

Figure 5 shows the three motors positioned at a distance R from the origin, (*i.e.*, the base center), and placed at equal angles to each other, where the angle between the wheel axes is 120 degrees. The angles *θ_1_*, *θ_2_*, and *θ_3_* are the angles of the wheels measured relative to the x y plane. *φ* is the rotational angle of the robot; *V_1_*, *V_2_*, and *V_3_* represent the three wheel speeds, and *V_m_* is the target movement direction. The experimental setup of the robot is shown in Figure 6.

*V_m_* is the intended movement direction of the robot and can be represented as *V_m_* = (*V_mx_*, *V_my_*), form its x and y components. *φ* is the robot's rotate angular velocity. The equations of motion *V_1_*, *V_2_*, *V_3_* are as expressed as:

(1) $$\left[\begin{array}{c} V_{1}\\ V_{2}\\ V_{3} \end{array}\right]=\left[\begin{array}{ccc} \cos\theta_{1} & \sin\theta_{1} & R\\ \cos\theta_{2} & \sin\theta_{2} & R\\ \cos\theta_{3} & \sin\theta_{3} & R \end{array}\right]\;\left[\begin{array}{c} V_{mx}\\ V_{my}\\ \dot{\phi } \end{array}\right]$$

**A** movement rule base can be designed from the kinematic equation.

### 3.2. Fuzzy Controller Design from Motor Encoders

Robots in real environments are easily affected by several factors. For example, the output power of the three groups of motors may be uneven, ground friction may vary, or the weight balance may be uneven. All these factors can cause the path of the robot to deviate.

**Figure 7.**
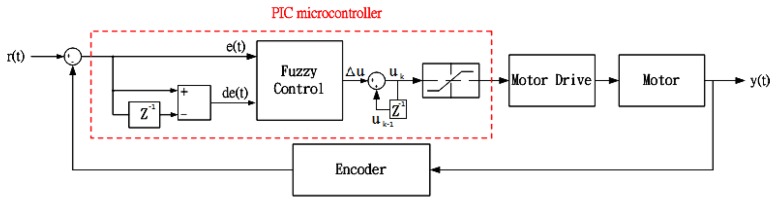
The control system block diagram.

As shown in Figure 7, the output the output *u_k_* is the sum of *u_k-1_* and Δ*u*. Δ*u* is the motor revolution error and error difference, represents the amount of PWM adjustment needed. The inputs to the fuzzy controller are *e* and *de*, where *e* is motor speed error and *de* is error variation. Therefore, the straight path deviation is compensated through an invariant motor revolution.

**Figure 8.**
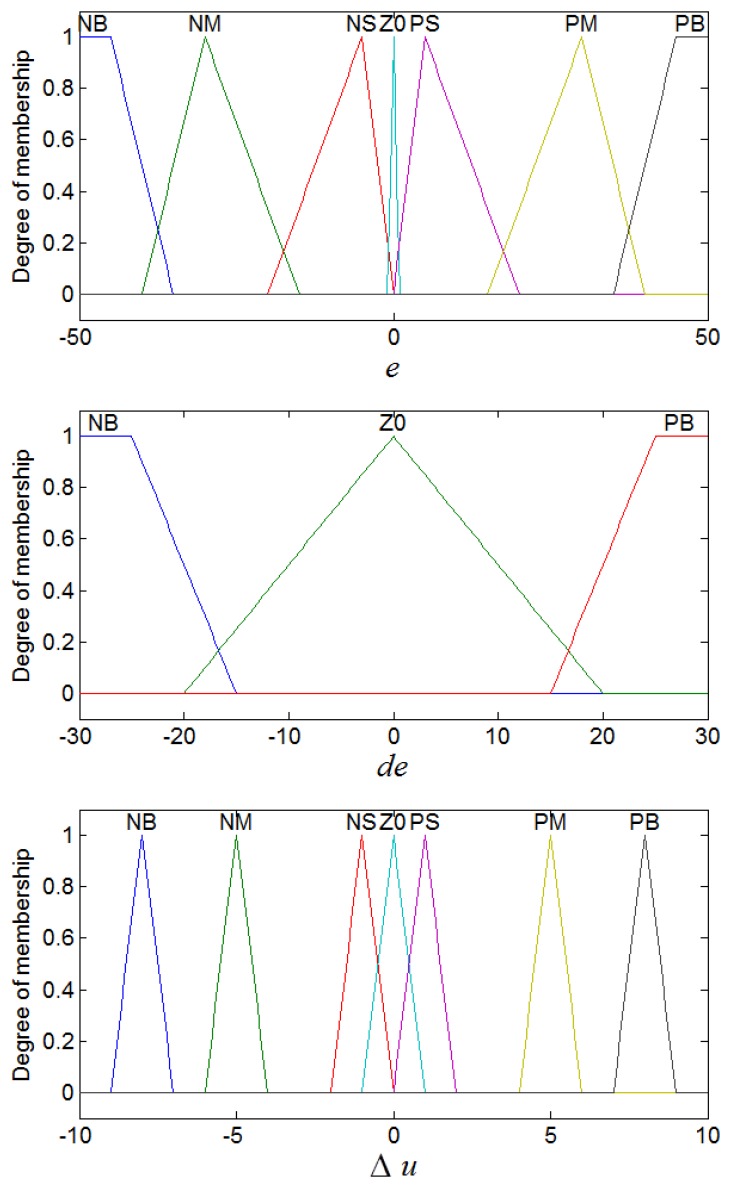
Input and output membership functions.

Figure 8 shows membership functions of the input variable *e* and *de* and the output Δ*u*, where a triangle function is used as the single membership function. The fuzzy set is composed of the following values: negative big (NB), negative medium (NM), negative small (NS), zero (ZO), positive small (PS), positive medium (PM) and positive big (PB).

By developing rules of thumb based upon several measurements, premise and consequence are deduced and the tuned ranges of membership functions are determined accordingly. The rule base consisting of 21 rules for motor encoder compensation is given in Table 1, and an example is illustrated by:

(2) If e is NM and de is ZO,thenΔu is PM.

It is assumed that if the motor revolution error e is NM, the revolution error difference de is ZO, and the PWM adjustment (Δ*u*) is PM. This situation is assumed to represent a realistic motor revolution value that is less than the expected value, which has an error within the tolerance range. Therefore, the motor PWM is increased by the value of PM.

**Figure 9.**
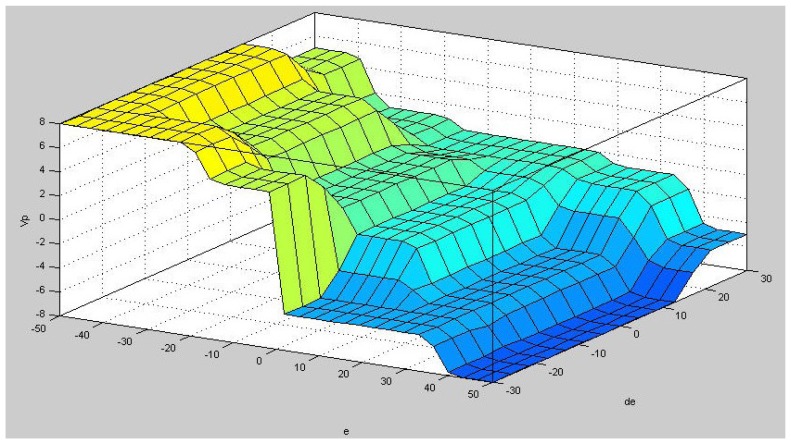
The fuzzy input and output relationship graph.

We perform a fuzzy interference using a minimum inference engine. The controller output represents the center of gravity defuzzification and is determined by the algorithm. The input and output relationship curve of the fuzzy controller is shown in Figure 9.

**Figure 10.**
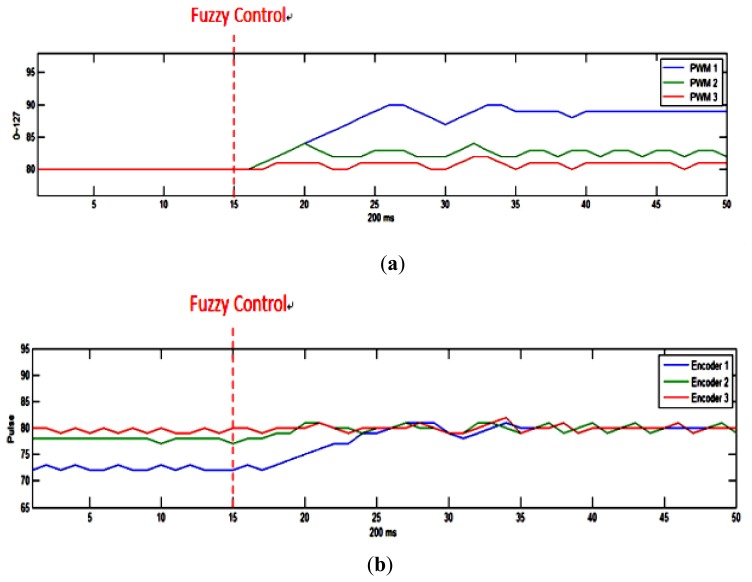
Experimental results of the motor revolution using fuzzy theory. (**a**) PWM tuning curves and (**b**) encoder feedback curves for the three motor sets.

We set motor PWM to divide into 128 equal parts, *i.e.*, 0–127. As shown in Figure 10(a), we set the initial values of the PWM driving the three motors to 80. A straight path deviation is observed because of the non-uniform feedback signal. Figure 10(b) shows the activation of the fuzzy controller at 15ms to equalize the three motor revolutions. However, it should be noted that the three motor sets experience different levels of PWM due to variation in the load carried by the robot, motor efficiency, and other factors.

## 4. An Extensible Obstacle-Avoidance System Design

**Figure 11.**
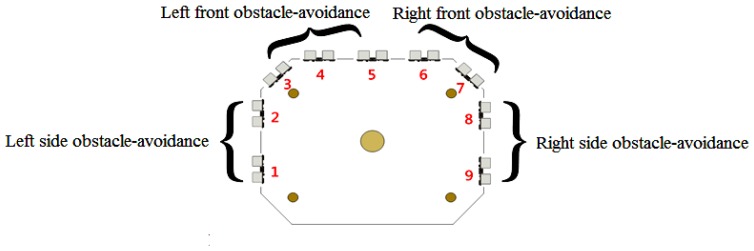
Ultrasonic distance sensors shown on the robot periphery.

Extension theory is used to describe the inference process of obstacle-avoidance, which allows us to transform a complex problem in the real world into one expressed through matter-elements. As shown in Figure 11 and in Equation (3), nine sets of ultrasonic distance sensors are installed on the left, front-left, front-right, and right sides. An appropriate movement path can be selected by applying extension theory after converting the analogue distance signal into a digital value:

(3) $$\{\begin{array}{ll} \text{Left side distance} & =\text{Ultrasonic}\;1\cap \text{Ultrasonic}\;2\\ \text{Left front distance} & =\text{Ultrasonic}\;3\cap \text{Ultrasonic}\;4\cap \text{Ultrasonic}\;5\\ \text{Right front distance} & =\text{Ultrasonic}\;6\cap \text{Ultrasonic}\;7\\ \text{Right side distance} & =\text{Ultrasonic}\;8\cap \text{Ultrasonic}\;9 \end{array}$$

### 4.1. Matter-Element Extension Set

In this section we quantify the extension set characteristics mathematically. The set with name (Name, N), characteristic (Characteristic, C), and with characteristic value (Value, V) is used to describe the three basic elements.

In this work, the obstacle-avoidance system is modeled as multi-dimensional obstacle-avoidance matter-elements, where the names of the extension matter-elements are the same as the number of obstacle-avoidance modes. The ultrasonic distance sensors are defined by four characteristics. Various motion approaches based on the principle of the multi-dimensional extension matter-element model, are expressed in Equation (4). These are associated with various motion strategies:

(4) $$\{\begin{array}{llll} R_{1,i1}= & \left[\begin{array}{ccc} N_{1,i1} & ,C_{1,i1,j} & ,<a_{1,i1,j},b_{1,i1,j}> \end{array}\right] & ,i1=1,2,\cdots ,7 & ,j=1,2,3,4\\ R_{2,i2}= & \left[\begin{array}{ccc} N_{2,i2} & ,C_{2,i2,j} & ,<a_{2,i2,j},b_{2,i2,j}> \end{array}\right] & ,i2=1,2,\cdots ,6 & ,j=1,2,3,4\\ R_{3,i3}= & \left[\begin{array}{ccc} N_{3,i3} & ,C_{3,i3,j} & ,<a_{3,i3,j},b_{3,i3,j}> \end{array}\right] & ,i3=1,2,\cdots ,6 & ,j=1,2,3,4 \end{array}$$

*R_1,i1_*, *R_2,i2_*, *R_3,i3_* represent various matter-element models, *N_1,i1_*, *N_2,i2_*, *N_3,i3_* are the names of various obstacle modes, and *C_1,i1_*, *C_2,i2_*, *C_3,i3_* are the distances to the obstacles in each aspect. The terms <*a_1,i1,j_*, *b_1,i1,j_*>, <*a_2,i2,j_*, *b_2,i2,j_*>, <*a_3,i3,j_*, *b_3,i3,j_*> are the scopes of the classical domains defined in various aspects. This work comprises three sets of models, representing various motion approaches. Strategies are built to avoid obstacles in various aspect directions. There are up to seven motion strategies specified for the forward motion, and six for the left forward and the right forward, as show in Tables 2 to 4.

### 4.2. Correlation Function

**Figure 12.**
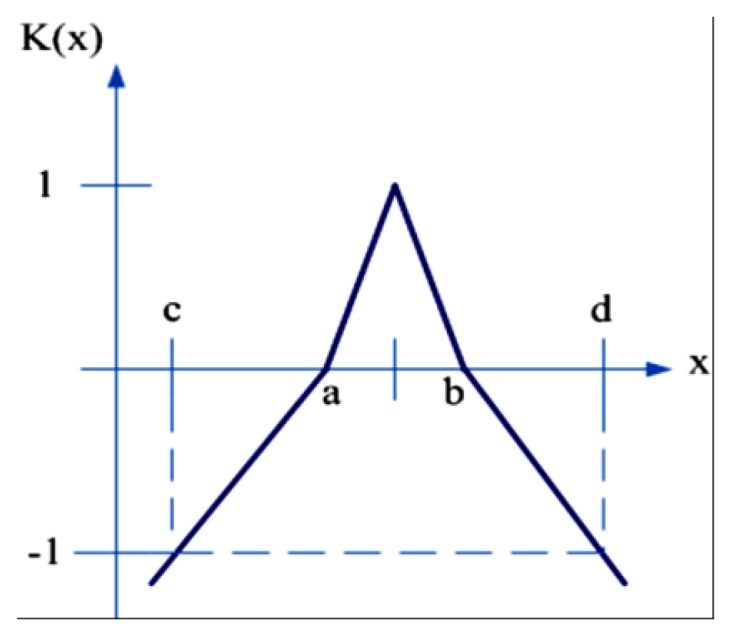
An extension correlation function.

In Figure 12 a classical domain and a neighborhood domain are defined on the interval *X_0_* = <*a*, *b*> and *X* = <*c*, *d*> respectively. The neighborhood domain is defined as *X* = <0, 200>, and *X_0_ X*, without any common end points. The primary correlation function can be defined as:

(5) $$K(x)=\frac{\rho (x,X_{O})}{D(x,X_{O},X)}$$

where *D*(*x*, *X_0_*, *X*) is a point position value, and the relationship between a point and two different ranges is defined as:

(6) $$D(x,X_{O},X)=\{\begin{array}{cc} \rho (x,X)-\rho (x,X_{O}) & ,\text{x}\notin {\text{X}}_{\text{O}}\\ -1 & ,\text{x}\epsilon {\text{X}}_{\text{O}} \end{array}$$

The relationship between x and *X*_0_ is defined as:

(7) $$\rho (x,X_{O})=\left|x-\frac{a+b}{2}\right|-\frac{1}{2}(b-a)$$

where the point *x* is related to the range *X* as:

(8) $$\rho (x,X)=\left|x-\frac{c+d}{2}\right|-\frac{1}{2}(d-c)$$

### 4.3. Evaluation Method and the Best Strategy of Obstacle Avoidance

**Figure 13.**
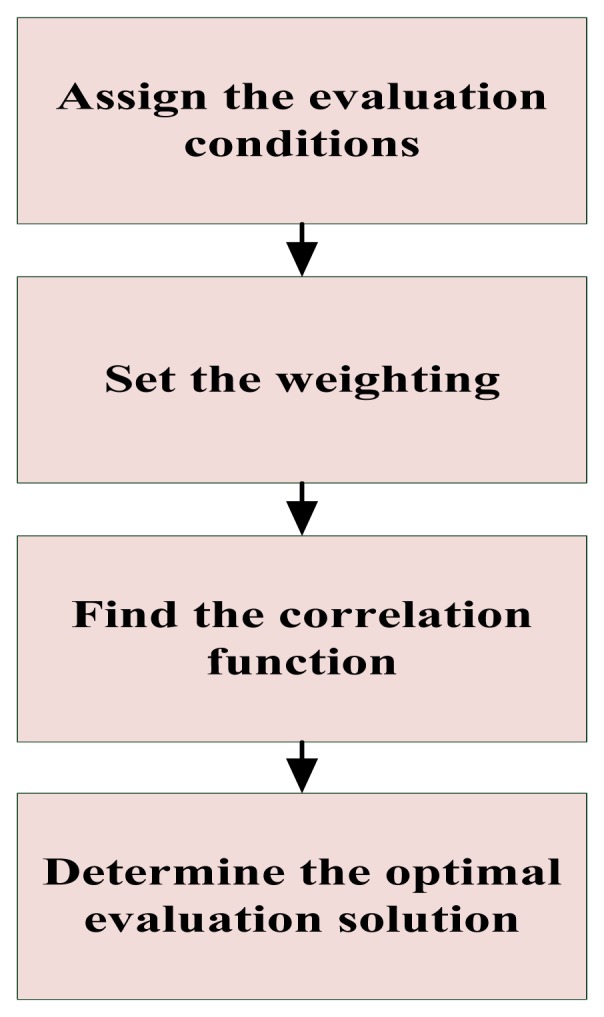
The procedure for determining an optimal evaluation strategy.

In Figure 13 the procedure for an optimal evaluation strategy is described in order to find the best strategy of obstacle-avoidance. The optimal obstacle-avoidance depends on the obtained maximum correlation degree by means of the following procedure:

1. We first define evaluation conditions. The correlation set *K_1,i1_*, *K_2,i2_*, *K_3,i3_*, (*i.e.*, the obstacle-avoidance strategies) made by three mobile modes and four sets of distance sensors mounted in various angles, are expressed as:
  (9) $$\{\begin{array}{ccc} K_{1,i1}=\{K_{1,i1,j}\} & i1=1,2,\cdots ,7 & ,j=1,2,3,4\\ K_{2,i2}=\{K_{2,i2,j}\} & i2=1,2,\cdots ,6 & ,j=1,2,3,4\\ K_{3,i3}=\{K_{3,i3,j}\} & i3=1,2,\cdots ,6 & ,j=1,2,3,4 \end{array}$$
2. The weightings *W_1,i1,j_*, *W_2,i2,j_*, *W_3,i3,j_* of four sets of distance sensors to detect obstacles are assigned the same value of 1/4. The correlation between each distance sensed is expressed as:
  (10) $$\{\begin{array}{ll} {\bar{K}}_{1,i1}=\sum_{j=1}^{4}W_{1,i1,j}K_{1,i1,j} & ,\text{i}1=1,2,\ldots ,7\\ {\bar{K}}_{2,i2}=\sum_{j=1}^{4}W_{2,i2,j}K_{2,i2,j} & ,\text{i}2=1,2,\ldots ,6\\ {\bar{K}}_{3,i3}=\sum_{j=1}^{4}W_{3,i3,j}K_{3,i3,j} & ,\text{i}3=1,2,\ldots ,6 \end{array}$$
3. The maximum degree of correlation in the individual mobile modes is extracted for the optimal obstacle-avoidance strategy, and can be found by comparing the optimal degree of evaluation between *K_1,i1_*, *K_2,i2_* and *K_3,i3_*, as:
  (11) $$\{\begin{array}{c} {\bar{K}}_{1\max}=\underset{i1=1,2,\cdots 7}{\max}{\bar{K}}_{1,i1}\\ {\bar{K}}_{2\max}=\underset{i2=1,2,\cdots 6}{\max}{\bar{K}}_{2,i2}\\ {\bar{K}}_{3\max}=\underset{i3=1,2,\cdots 6}{\max}{\bar{K}}_{3,i3} \end{array}$$
4. The choice of obstacle-avoidance mode for a robot is evaluated as the degree of correlation within a set of multi-dimensional obstacle-avoidance matter-element modes. This is then translated into the optimal strategy to avoid obstacles.

In Equation (12) the directions representing the mobile mode are given. If the direction is equal to 1, it denotes forward motion, 2 represents front forward, and any other value represents a forward-right direction. The obstacle direction representing the optimal obstacle-avoidance strategy is sent to the robot, where *K_1__max* represents the optimal strategy for forward motion, *K_2__max* for forward-left motion, and *K_3__max* for forward-right:

(12) $$\begin{array}{l} if(\text{direction}==1)\text{obstacle}\_\text{direction}=K1\_\max;\\ \text{elseif}(\text{direction}==2)\text{obstacle}\_\text{direction}=K2\_\max;\\ \text{elseobstacle}\_\text{direction}=K3\_\max; \end{array}$$

## 5. Experimental Results and Analysis

**Figure 14.**
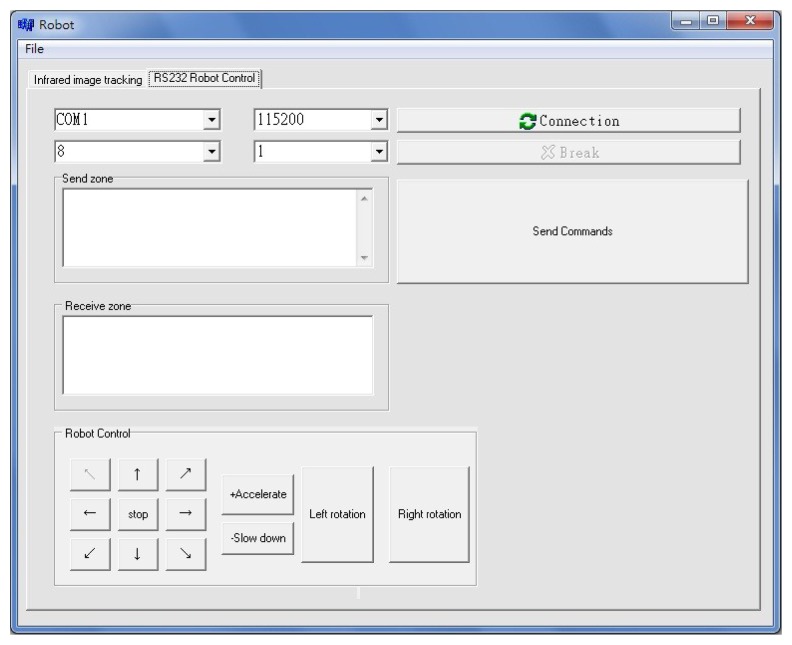
The data link control interface.

**Figure 15.**
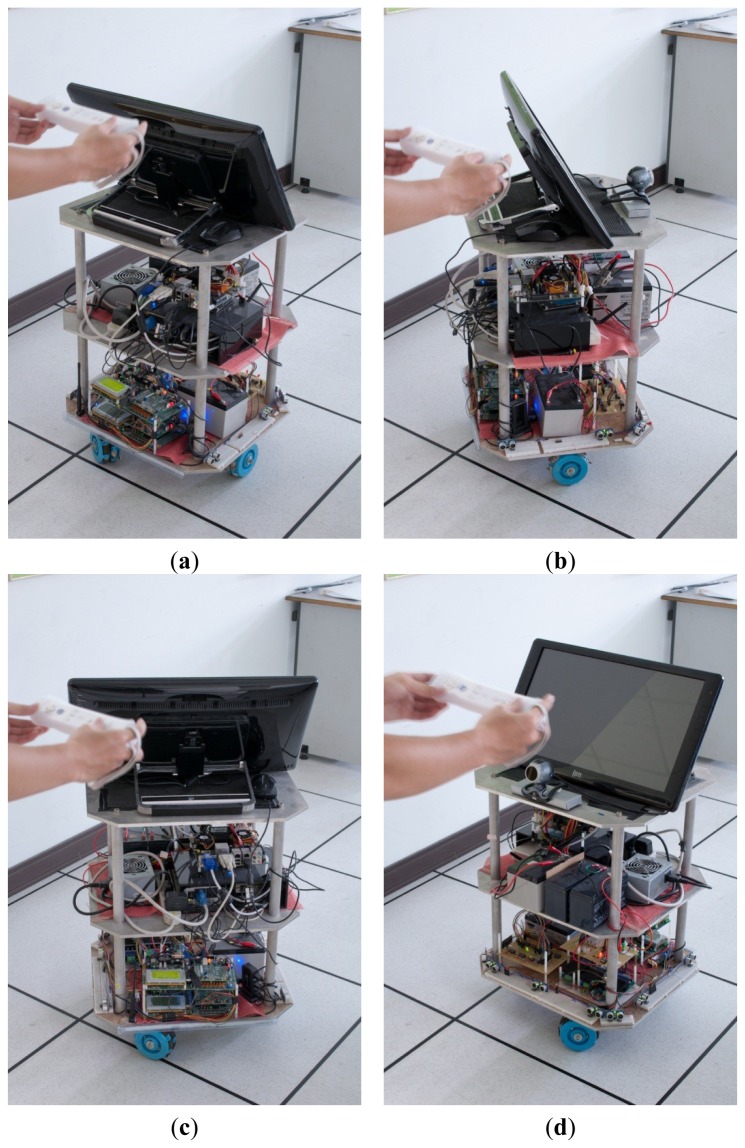
A set of omni-directional mobile experiments. (**a**) Forward, (**b**) turn right, (**c**) turn left, and (**d**) rotate back.

**Figure 16.**
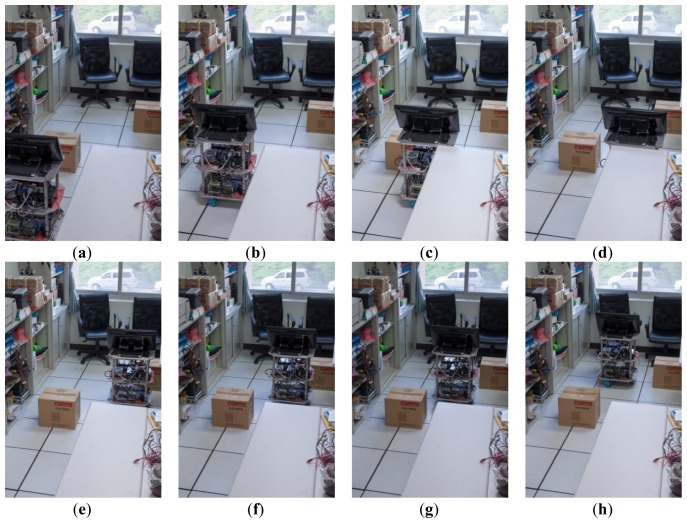
A set of obstacle-avoidance experiments. (**a**–**d**) Actual experimental pictures of the robot pass the first obstacle and (**e**–**h**) actual experimental pictures of the robot pass the second obstacle.

The interface design of the autonomous mobile robot controller is shown in Figure 14. A data link interface to the robot through an RS232 serial transmission, carries the control signal sent from the workstation to the micro controller. This is usually in manual mode. An operational test for obstacle-avoidance by the omni-directional robot was conducted, as shown in Figure 15 and Figure 16.

The omni-directional mobile robot was seen to exhibit high mobility in a complex environment. We also tested the obstacle avoidance capability. In order to correct the path deviation, the aspect angle of movement, and the target speed are transmitted to the three motor sets driven by a fuzzy logic controller to provide a compensation approach for the motor encoder. The robot was able to avoid all obstacles, proving the effectiveness of the proposed system. Thus, the feasibility and the effectiveness of the robot were validated.

## 6. Conclusions

In this study, omni-directional wheels were used to develop a robot capable of omni-directional movement. The robot offers improved mobility as it utilizes lateral movement over rotational movement by utilizing the omni-directional wheel design. The robot was tested in various mobile modes in a complex environment, and was able to compensate for path deviations through motor encoder compensation based on fuzzy logic theory. The robot was also able to avoid all obstacles in its path autonomously by employing ultrasonic distance sensors with an obstacle-avoidance algorithm. The aim of implementing omni-directional motion control for a three-wheeled autonomous robot was achieved. The robot offers high mobility, motion path correction, and an obstacle avoidance capability. This robot system is suitable for libraries, supermarkets, airports, hospitals and similar scenarios.

## Figures and Tables

**Table 1.** A motor encoder compensation rule table.

***e***	**NB**	**NM**	**NS**	**ZO**	**PS**	**PM**	**PB**

***de***							
NB	PB	PB	PM	ZO	NM	NM	NB
ZO	PB	PM	PS	ZO	NS	NM	NB
PB	PM	PS	ZO	ZO	ZO	NS	NM

**Table 2.** Rule table for forward motion in the extension-element model.

**Number**	**Obstacle Location**	**Extension Element Model**	**Approach**
1	No obstacle	${\text{R}}_{11}=\left[\begin{array}{lll} N_{11} & ,C_{111} & ,<15,200>\\ & ,C_{112} & ,<15,200>\\ & ,C_{113} & ,<15,200>\\ & ,C_{114} & ,<15,200> \end{array}\right]$	Move forward
2	Left forward	${\text{R}}_{12}=\left[\begin{array}{lll} N_{12} & ,C_{121} & ,<15,200>\\ & ,C_{122} & ,<0,15>\\ & ,C_{123} & ,<15,200>\\ & ,C_{124} & ,<15,200> \end{array}\right]$	Move right
3	Right forward	${\text{R}}_{13}=\left[\begin{array}{lll} N_{13} & ,C_{131} & ,<15,200>\\ & ,C_{132} & ,<15,200>\\ & ,C_{133} & ,<0,15>\\ & ,C_{134} & ,<15,200> \end{array}\right]$	Move left
4	Left forward, Right forward	${\text{R}}_{14}=\left[\begin{array}{ccc} N_{14} & ,C_{141} & ,<15,200>\\ & ,C_{142} & ,<0,15>\\ & ,C_{143} & ,<0,15>\\ & ,C_{144} & ,<15,200> \end{array}\right]$	Move left or right
5	Left forward, Right forward, Right	${\text{R}}_{15}=\left[\begin{array}{lll} N_{15} & ,C_{151} & ,<15,200>\\ & ,C_{152} & ,<0,15>\\ & ,C_{153} & ,<0,15>\\ & ,C_{154} & ,<0,15> \end{array}\right]$	move left
6	Left forward, Right forward, Left	${\text{R}}_{16}=\left[\begin{array}{lll} N_{16} & ,C_{161} & ,<0,15>\\ & ,C_{162} & ,<0,15>\\ & ,C_{163} & ,<0,15>\\ & ,C_{164} & ,<15,200> \end{array}\right]$	Move right
7	All	${\text{R}}_{17}=\left[\begin{array}{lll} N_{17} & ,C_{171} & ,<0,15>\\ & ,C_{172} & ,<0,15>\\ & ,C_{173} & ,<0,15>\\ & ,C_{174} & ,<0,15> \end{array}\right]$	Move backward

**Table 3.** Rule table for left forward motion in the extension-element model.

**Number**	**Obstacle Location**	**Extension Element Model**	**Approach**
1	No obstacle	${\text{R}}_{21}=\left[\begin{array}{lll} N_{21} & ,C_{211} & ,<15,200>\\ & ,C_{212} & ,<15,200>\\ & ,C_{213} & ,<15,200>\\ & ,C_{214} & ,<15,200> \end{array}\right]$	Move left forward
2	Left	${\text{R}}_{22}=\left[\begin{array}{lll} N_{22} & ,C_{221} & ,<0,15>\\ & ,C_{222} & ,<15,200>\\ & ,C_{223} & ,<15,200>\\ & ,C_{224} & ,<15,200> \end{array}\right]$	Move forward
3	Left, Left forward	${\text{R}}_{23}=\left[\begin{array}{lll} N_{23} & ,C_{231} & ,<0,15>\\ & ,C_{232} & ,<0,15>\\ & ,C_{233} & <15,200>\\ & ,C_{234} & ,<15,200> \end{array}\right]$	Move right
4	Left, Right forward	${\text{R}}_{24}=\left[\begin{array}{lll} N_{24} & ,C_{241} & ,<0,15>\\ & ,C_{242} & ,<15,200>\\ & ,C_{243} & ,<0,15>\\ & ,C_{244} & ,<15,200> \end{array}\right]$	Move right
5	Left, Left forward, Right forward	${\text{R}}_{25}=\left[\begin{array}{lll} N_{25} & ,C_{251} & ,<0,15>\\ & ,C_{252} & ,<0,15>\\ & ,C_{253} & ,<0,15>\\ & ,C_{254} & ,<15,200> \end{array}\right]$	Move right
6	Upper left corner	${\text{R}}_{26}=\left[\begin{array}{lll} N_{26} & ,C_{261} & ,<15,200>\\ & ,C_{262} & ,<0,15>\\ & ,C_{263} & ,<15,200>\\ & ,C_{264} & ,<15,200> \end{array}\right]$	Move right forward

**Table 4.** Rule table for right forward motion in the extension-element model.

**Number**	**Obstacle Location**	**Extension Element Model**	**Approach**
1	No obstacle	${\text{R}}_{31}=\left[\begin{array}{lll} N_{31} & ,C_{311} & ,<15,200>\\ & ,C_{312} & ,<15,200>\\ & ,C_{313} & ,<15,200>\\ & ,C_{314} & ,<15,200> \end{array}\right]$	Move right forward
2	Right	${\text{R}}_{32}=\left[\begin{array}{lll} N_{32} & ,C_{321} & ,<15,200>\\ & ,C_{322} & ,<15,200>\\ & ,C_{323} & ,<15,200>\\ & ,C_{324} & ,<0,15> \end{array}\right]$	Move forward
3	Left forward, Right	${\text{R}}_{33}=\left[\begin{array}{lll} N_{33} & ,C_{331} & ,<15,200>\\ & ,C_{332} & ,<0,15>\\ & ,C_{333} & <15,200>\\ & ,C_{334} & ,<0,15> \end{array}\right]$	Move left
4	Right forward, Right	${\text{R}}_{34}=\left[\begin{array}{lll} N_{34} & ,C_{341} & ,<15,200>\\ & ,C_{342} & ,<15,200>\\ & ,C_{343} & ,<0,15>\\ & ,C_{344} & ,<0,15> \end{array}\right]$	Move left
5	Left forward, Right forward, Right	${\text{R}}_{35}=\left[\begin{array}{lll} N_{35} & ,C_{351} & ,<15,200>\\ & ,C_{352} & ,<0,15>\\ & ,C_{353} & ,<0,15>\\ & ,C_{354} & ,<0,15> \end{array}\right]$	Move left
6	Upper right corner	${\text{R}}_{36}=\left[\begin{array}{lll} N_{36} & ,C_{361} & ,<15,200>\\ & ,C_{362} & ,<15,200>\\ & ,C_{363} & ,<0,15>\\ & ,C_{364} & ,<15,200> \end{array}\right]$	Move left forward
